# Hormonal Responses to *Plasmodiophora brassicae* Infection in *Brassica napus* Cultivars Differing in Their Pathogen Resistance

**DOI:** 10.3390/ijms19124024

**Published:** 2018-12-13

**Authors:** Sylva Prerostova, Petre I. Dobrev, Veronika Konradyova, Vojtech Knirsch, Alena Gaudinova, Barbara Kramna, Jan Kazda, Jutta Ludwig-Müller, Radomira Vankova

**Affiliations:** 1 Institute of Experimental Botany Czech Acad Sci, Laboratory of Hormonal Regulations in Plants, Rozvojova 263, 165 02 Prague 6, Czech Republic; prerostova@ueb.cas.cz (S.P.); dobrev@ueb.cas.cz (P.I.D.); knirsch@ueb.cas.cz (V.K.); gaudinova@ueb.cas.cz (A.G.); kramna@ueb.cas.cz (B.K.); 2 Faculty of Agrobiology, Food and Natural Resources, Department of Plant Protection, Czech University of Life Sciences Prague, Kamycka 129, 165 00 Prague 6, Czech Republic; konradyova@af.czu.cz (V.K.); kazda@af.czu.cz (J.K.); 3 Department of Experimental Plant Biology, Charles University, Faculty of Science, Vinicna 5, 128 44 Prague 2, Czech Republic; 4 Dresden, Faculty of Biology, Institute of Botany, Technische Universität, 01062 Dresden, Germany; jutta.ludwig-mueller@tu-dresden.de

**Keywords:** auxin, *Brassica napus*, cytokinin, gene expression, jasmonic acid, *Plasmodiophora brassicae*, plant hormone, resistance, salicylic acid

## Abstract

Hormonal dynamics after *Plasmodiophora brassicae* infection were compared in two *Brassica napus* cultivars—more resistant SY Alister and more sensitive Hornet, in order to elucidate responses associated with efficient defense. Both cultivars responded to infection by the early transient elevation of active cytokinins (predominantly *cis*-zeatin) and auxin indole-3-acetic acid (IAA) in leaves and roots, which was longer in Hornet. Moderate IAA levels in Hornet roots coincided with a high expression of biosynthetic gene *nitrilase* *NIT1* (contrary to *TAA1*, *YUC8*, *YUC9*). Alister had a higher basal level of salicylic acid (SA), and it stimulated its production (via the expression of *isochorismate synthase* (*ICS1*)) in roots earlier than Hornet. Gall formation stimulated cytokinin, auxin, and SA levels—with a maximum 22 days after inoculation (dai). SA marker gene *PR1* expression was the most profound at the time point where gall formation began, in leaves, roots, and especially in galls. Jasmonic acid (JA) was higher in Hornet than in Alister during the whole experiment. To investigate SA and JA function, SA was applied before infection, and twice (before infection and 15 dai), and JA at 15 dai. Double SA application diminished gall formation in Alister, and JA promoted gall formation in both cultivars. Activation of SA/JA pathways reflects the main differences in clubroot resistance.

## 1. Introduction

The obligate biotroph *Plasmodiophora brassicae* may cause clubroot disease in 3700 members of the family *Brassicaceae* [1], including economically important oilseed rape (canola), broccoli, cabbage, and cauliflower. The biotrophic lifestyle is characterized by two phases. The primary phase occurs in root hairs of infected plants. The secondary phase takes place in the cortex and stele of hypocotyl and roots and is associated with cell hypertrophy (cell division) and hyperplasia (cell elongation) resulting in gall formation [2]. Haploid resting spores are formed in galls. After gall disintegration, spores can survive in the soil for up to 17 years [3]. The longevity of resting spores in the soil strongly contributes to the gradual spreading of *P. brassicae* infection in Europe and North America (including Canada [1]), which makes this pathogen one of the most serious threats for *Brassicaceae* cultivation. Up to now, chemical treatments (e.g., with sodium N-methyldithiocarbamate) have had rather limited effects [4]. For this reason, cultivation of (at least partially) resistant cultivars represents the only effective solution. The understanding of the mechanisms of efficient plant defense represents an important prerequisite for the breeding of new resistant cultivars. 

Infection development requires the modulation of host metabolism, including hormone pools [5]. Gall formation is associated with the stimulation of cell division, which requires cytokinin (CK) and auxin activity. Moreover, CKs enhance the sink strength; thus their elevation in galls is important for the attraction of nutrients to infected roots, as the pathogen is dependent on nutrients from the host, such as carbohydrates, amino acids, and lipids [5,6]. Enhanced CK levels in roots after *P. brassicae* infection were reported already by Dekhuijzen and Overeem [7] or by Dekhuijzen [8]. Müller and Hilgenberg [9] demonstrated that *P. brassicae* is able to convert adenine to *trans*-zeatin and its riboside, which indicates that the pathogen is able to form CKs at least in limited amounts. Further evidence for the involvement of CKs came from Siemens et al. [10], who showed that CK-depleted *Arabidopsis thaliana* plants were more tolerant to the clubroot pathogen. When the early response of *A. thaliana* to *P. brassicae* infection was followed [11], plasmodial-produced CKs (mainly isopentenyladenine and isopentenyladenosine) triggered the CK response (expression of response regulator *ARR5*) three days after inoculation (dai), which resulted in the local re-initiation of cell division in the root cortex 4 dai. Even if during the later stages CKs were not differentially regulated in *A. thaliana*, it was suggested that they could also regulate the development of the plasmodial stage of *P. brassicae* [12].

At 6 dai, elevation of auxin (indole-3-acetic acid, IAA) content, as well as the stimulation of auxin-inducible genes was observed, promoting morphological changes in the roots including cell elongation. Polar auxin transport seems to be indispensable for early clubroot symptom development [11]. After early up-regulation, auxin content may be maintained, even at the later stages of infection [5]. The up-regulation of nitrilases and myrosinases seems to be responsible for this effect via the indole-3-acetonitrile pathway [5,13]. The other growth-promoting hormones that were reported to be involved in *P. brassicae* infection are brassinosteroids [14].

The pathogen imposes alteration of the levels of other hormones, too. Abscisic acid (ABA), hormone predominantly associated with abiotic stress responses, was found to be elevated in the host plant at the later stages of *P. brassicae* infection, at least partially due to the restriction of water transport by roots and the resulting water deficit in the above-ground parts [5].

The key hormone in the defense against biotroph infection is salicylic acid (SA). Its level was found to be strongly enhanced by *P. brassicae* infection in resistant Bur-0 ecotype of *A. thaliana* [15], in contrast to an only mild elevation of jasmonic acid (JA) levels and signaling pathway. A substantial JA increase was detected in Bur-0 after infection with another, more aggressive pathotype [16], as well as in the sensitive ecotype *A. thaliana* Col-0 [15]. Furthermore, mutants with a constitutive SA-response and *PR1*-expression (*cpr1*) showed enhanced resistance to *P. brassicae* [17].

Elevation of JA biosynthesis and JA-responsive gene expression in sensitive Col-0 may raise a question whether this effect is a part of the host plant defense or a part of the pathogen strategy to overcome plant defense by suppression of the SA pathway. A similar effect was observed in the case of coronatine produced by *Pseudomonas syringae* [18].

The pathogen responses may differ among different species or even cultivars (e.g., *A. thaliana* and *Brassica* species [5,19]). In this study, we have compared two cultivars of *Brassica napus*—the more resistant cultivar Alister and the sensitive cultivar Hornet—in order to characterize responses associated with enhanced resistance to *P. brassicae*. The responses have been determined at the levels of hormones (CK, auxin, SA, JA, and ABA) as well as the expression of hormone-related genes. Taking into account the communication between roots and shoots, the effect of *P. brassicae* infection was followed not only in roots and galls, but also in leaves.

## 2. Results

### 2.1. Plasmodiophora Brassicae Infection Responses Differ Between Cultivars and Hormone Treatments

**Figure 1:**
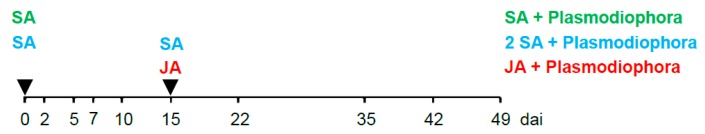
Scheme of the experiment. *Brassica napus* plants were inoculated (except the control variant) by *Plasmodiophora brassicae* 10 days after sowing. Part of the inoculated plants was treated (as marked by 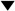) by foliar spray with 1 mM salicylic acid (SA)—once (SA + *Plasmodiophora*) or twice (two SA + *Plasmodiophora*); or with 1 mM jasmonic acid (JA) (JA + *Plasmodiophora*). Samples were collected 2–49 days after inoculation (dai) at the days indicated in the scheme.

Two *B. napus* cultivars with different resistance against *P. brassicae* pathotype 6 were analyzed in the respect to disease severity as well as to their responses to treatment with defense hormones. The cultivar SY Alister has been previously reported as resistant to *P. brassicae* infection [20]. The plants were treated by foliar spray with SA at the day of inoculation, or in the second set of experiments at the day of inoculation, and subsequently, 15 dai, which corresponded to the onset of gall formation. For the JA treatments, only one foliar spray at the later time point was performed (Figure 1).

The Disease Index (DI) expressing a degree of infestation was higher for the susceptible *B. napus* cultivar Hornet than for the resistant cultivar Alister for all variants in both years of the experiment (Table 1). The susceptible cultivar Hornet inoculated by *P. brassicae* had a DI of 100% each day of sampling in 2016. In 2017 the index increased from 40% at 22 dai to 100% at 49 dai. The DI of the resistant cultivar Alister varied from 7 to 40% in 2016, and from 0 to 13% in 2017.

Treatment of plants with SA and JA was carried out to determine if disease severity can be affected by hormone treatments. The SA-treated variants of Hornet had DI from 40 (22 dai after double SA application) to 87% (42 dai after single SA treatment) in both years. Both variants of Alister sprayed by SA had the DI between 7% and 13%. In the variants treated with JA, the DI for Hornet ranged from 56% 15 dai to 89% 42 dai; in the case of Alister, the DI reached 13% 22 dai. These data indicate that SA imposed a reduction of disease symptoms in the resistant cultivar, while JA application slightly promoted gall formation in both cultivars.

### 2.2. Growth-Promoting Hormones

**Figure 2:**
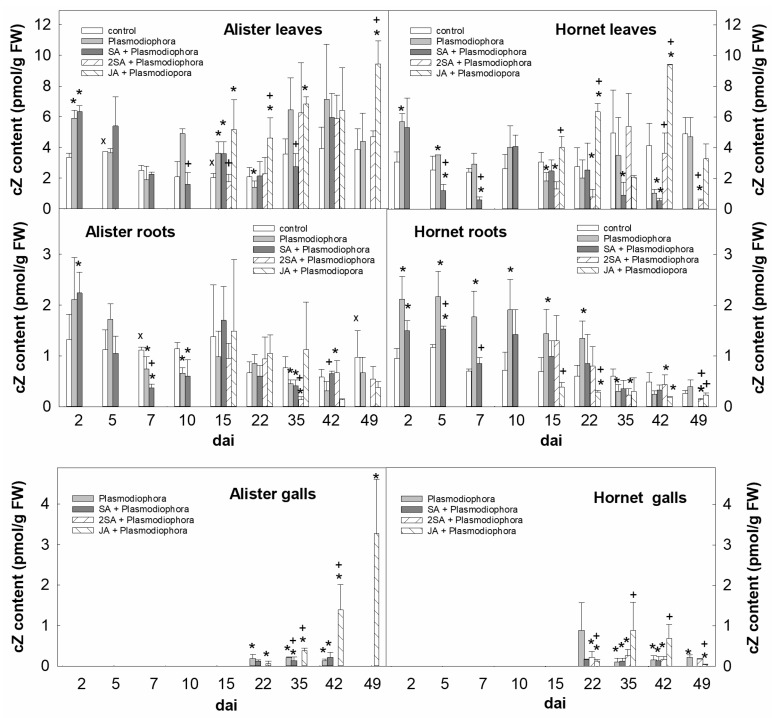
Levels of *cis*-zeatin (cZ) in leaves, roots, and galls of the sensitive cv. Hornet and the resistant cv. Alister. Control—non-infected plants, *Plasmodiophora*—plants infected by *P. brassicae*; SA + *Plasmodiophora*—infected plants treated by SA at the day of inoculation; 2SA + *Plasmodiophora*—infected plants treated twice by SA; JA + *Plasmodiophora*—infected plants treated by JA 15 dai. Statistically significant (two-sample Student’s *t*-test, *p* < 0.05, *n* = 4) sum of active CKs: * significant differences between the sample and control within the same time point; † significant differences between the sample treated by SA or JA and the variant inoculated by *Plasmodiophora brassicae* within the same time point; x significant differences between Alister and Hornet controls within the same time point. Mean values ± SD are shown in Table S2.

The levels of active CKs (*trans*-zeatin, dihydrozeatin, isopentenyladenine, and *cis*-zeatin) were followed in the interval 2–49 dai in leaves, roots and galls of the susceptible cultivar Hornet and the resistant cultivar Alister (detailed results on all active CKs are in Table S2). Early after infection (2 dai), the content of active CKs increased in leaves of both cultivars, mainly due to the increase of *cis*-zeatin (cZ, Figure 2). Slightly higher cZ levels were maintained in Hornet leaves within 10 dai, being decreased in Alister 5 and 7 dai. Application of 1 mM SA at the day of inoculation resulted in the decrease of cZ, which was immediate in Hornet leaves but delayed in leaves of Alister (Figure 2). Double SA application (before infection and 15 dai) had only short-term impact on active CKs. Application of 1 mM JA 15 dai promoted the production of cZ 15 and 22 dai in the leaves of both cultivars (Figure 2).

*P. brassicae* caused an elevation of the level of cZ between 2 and 10 dai in roots of Hornet, but only 2–5 dai in roots of Alister (Figure 2). The down-regulation of active CKs was found in roots of both cultivars at later time points (35–42 dai). The impact of a single SA application was observed in Hornet between 2–7 dai, in Alister from 7 dai. Double SA application had a negligible effect on active CKs in roots. JA application resulted in an increase of cZ in roots of both cultivars (since 15 dai, Figure 2). The levels of the most active CK in the stimulation of cell division—*trans*-zeatin—were elevated in galls, especially at 22 dai, being ca. four times higher in Hornet than in Alister. JA application enhanced *trans*-zeatin as well as isopentenyladenine levels in Hornet (35–42 dai), and *trans*-zeatin, cZ, and isopentenyladenine in Alister (35–49 dai) galls (Figure 2, Table S2).

**Figure 3:**
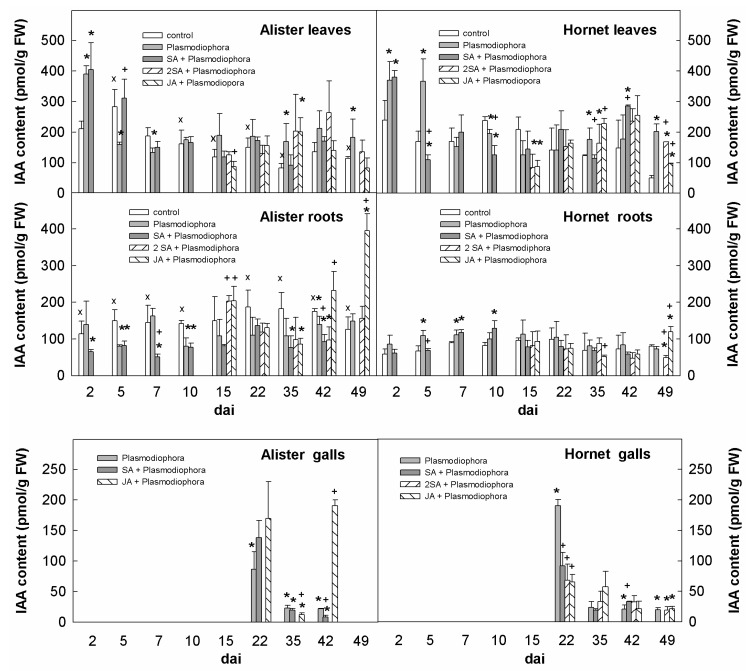
Levels of auxin indole-3-acetic acid (IAA) in leaves, roots, and galls of the sensitive cv. Hornet and the resistant cv. Alister. The description of variants is the same as in Figure 2. Statistically significant (two-sample Student’s *t*-test, *p* < 0.05, n = 4) sum of active CKs: * significant differences between the sample and control within the same time point; † significant differences between the sample treated by SA or JA and the variant inoculated by Plasmodiophora brassicae within the same time point; x significant differences between Alister and Hornet controls within the same time point.

*P. brassicae* infection was associated with an increase of IAA in Hornet leaves 2–5 dai, and in Alister 2 dai (Figure 3). A small elevation of IAA was found at the later stage of infection, in Hornet since 35 dai, and in Alister since 15 dai. SA applied 2 h before infection suppressed (with some delay) the impact of *P. brassicae* infection on auxin levels. The double SA, as well as the JA treatment, had negative effects on auxin levels close to their application (15 dai) in Hornet; but from 35 dai, auxin accumulation was stimulated. In Hornet and Alister roots, IAA was slightly enhanced up to 10 or 2 dai, respectively. SA application diminished this effect 2–5 dai in Hornet, and 2–7 dai in Alister. Both SA and JA slightly diminished IAA levels during the later stages of infection in Hornet roots. In Alister, JA exposure resulted in an increase of IAA content 42 and 49 dai (which coincided with gall formation). The beginning of gall formation (22 dai) caused by *P. brassicae* was characterized by a maximum IAA level in both cultivars.

### 2.3. Defence Hormones

**Figure 4:**
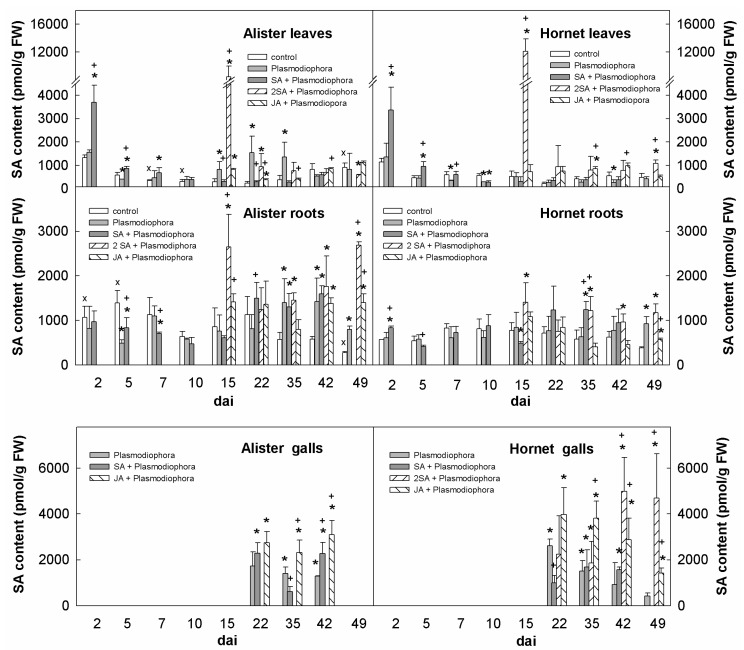
Levels of salicylic acid (SA) in leaves, roots, and galls of the sensitive cv. Hornet and the resistant cv. Alister. The description of variants is the same as in Figure 2. Statistically significant (two-sample Student’s *t*-test, *p* < 0.05, n = 4) sum of active CKs: * significant differences between the sample and control within the same time point; † significant differences between the sample treated by SA or JA and the variant inoculated by Plasmodiophora brassicae within the same time point; x significant differences between Alister and Hornet controls within the same time point.

The content of SA in Alister leaves was moderately increased during later time points (15–35 dai; Figure 4). Application of SA by foliar spray strongly enhanced the endogenous SA content in leaves of both cultivars. However, the effect was rather short-term, and significant down-regulation of SA was observed by 2 dai. An increase of SA levels in Hornet roots was found only during late infection time point (49 dai), and in Alister roots starting earlier (between 35–49 dai). Application of SA at the same day as the *P. brassicae* inoculation had a negligible impact on the endogenous SA content in roots after 2 days. A significant effect was observed after repeated SA treatments between 15–35 dai in Hornet, and a much stronger effect between 15–49 dai in Alister. *P. brassicae* infection was associated with elevation of SA levels in initiating galls of both cultivars (22 dai), with a descendent tendency in older galls. The double SA treatment promoted endogenous SA production in Hornet galls, especially between 42–49 dai. Exogenous JA had a positive impact on the SA content of galls in both cultivars.

**Figure 5:**
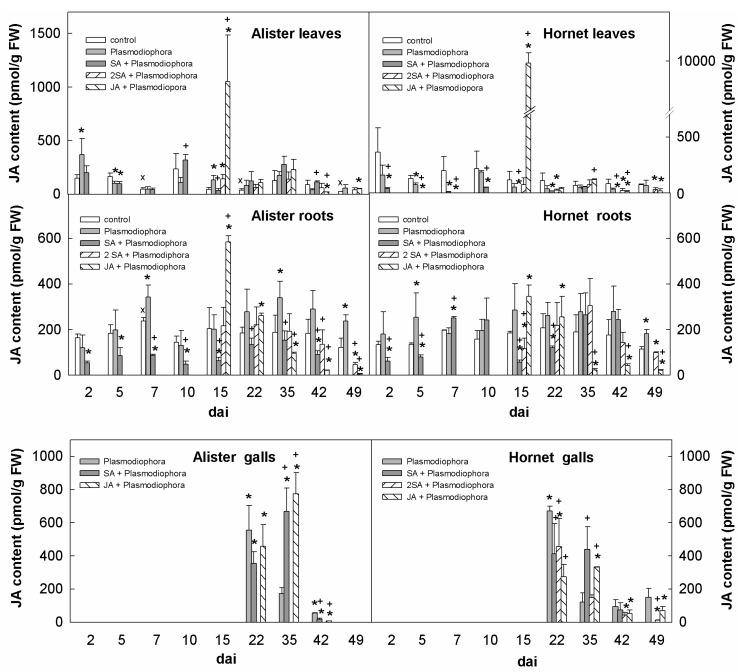
Levels of jasmonic acid (JA) in leaves, roots, and galls of the sensitive cv. Hornet and the resistant cv. Alister. The description of variants is the same as in Figure 2. Statistically significant (two-sample Student’s *t*-test, *p* < 0.05, n = 4) sum of active CKs: * significant differences between the sample and control within the same time point; † significant differences between the sample treated by SA or JA and the variant inoculated by Plasmodiophora brassicae within the same time point; x significant differences between Alister and Hornet controls within the same time point.

*P. brassicae* infection was generally associated with a decrease of JA in leaves, especially in Hornet (Figure 5). JA application led to the strong transient elevation of JA content in leaves at the day of foliar spray (15 dai). In contrast, the infected roots exhibited a JA promotion, maintained in Hornet for the whole experimental period. In Alister roots, slight JA down-regulation was found 2 dai and 10 dai, and a transient JA maximum was observed 7 dai. An increased JA content was found also during later stages of infection (15–49 dai), which was significant in Alister. SA application had a permanent negative effect on JA levels in Alister, and in Hornet only early after inoculation (2–5 dai), and at the beginning of gall formation (15–22 dai). JA application resulted in an elevation of endogenous JA in roots 15 dai, which was more profound in Alister than Hornet, decreasing quickly over the time. In both cultivars, young galls had the highest JA content at 22 dai.

### 2.4. Stress Hormones

**Figure 6:**
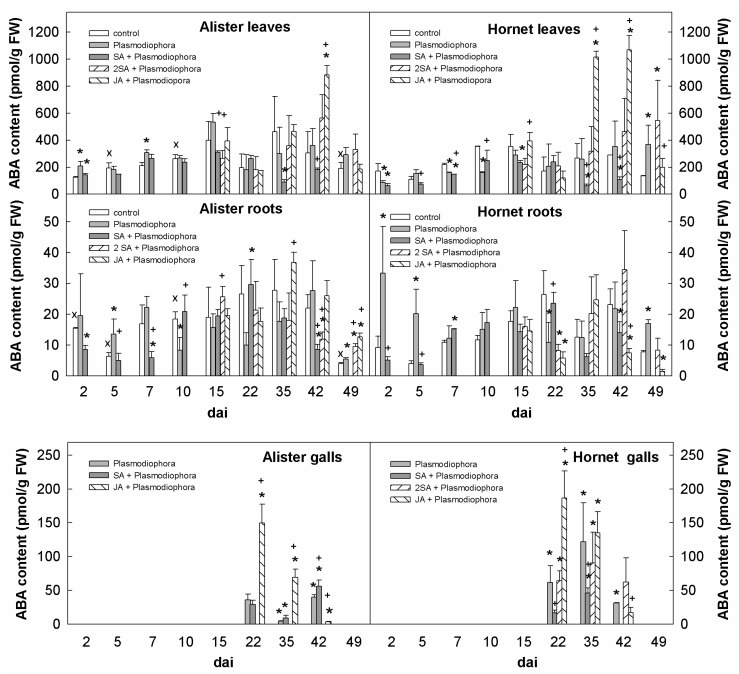
Levels of abscisic acid (ABA) in leaves, roots, and galls of the sensitive cv. Hornet and the resistant cv. Alister. The description of variants is the same as in Figure 2. Statistically significant (two-sample Student’s *t*-test, *p* < 0.05, n = 4) sum of active CKs: * significant differences between the sample and control within the same time point; † significant differences between the sample treated by SA or JA and the variant inoculated by Plasmodiophora brassicae within the same time point; x significant differences between Alister and Hornet controls within the same time point.

ABA levels better reflected the actual water relations than the effect of infection. ABA was much higher in leaves than in below-ground organs, showing a general elevation during later stages of the infection (Figure 6). JA application resulted in an increase of ABA levels in Alister galls at 22 and 35 dai, and in Hornet only during the earlier time point (22 dai).

### 2.5. Hormone-Related Gene Expression

#### 2.5.1. Cytokinin-Related Genes

**Figure 7:**
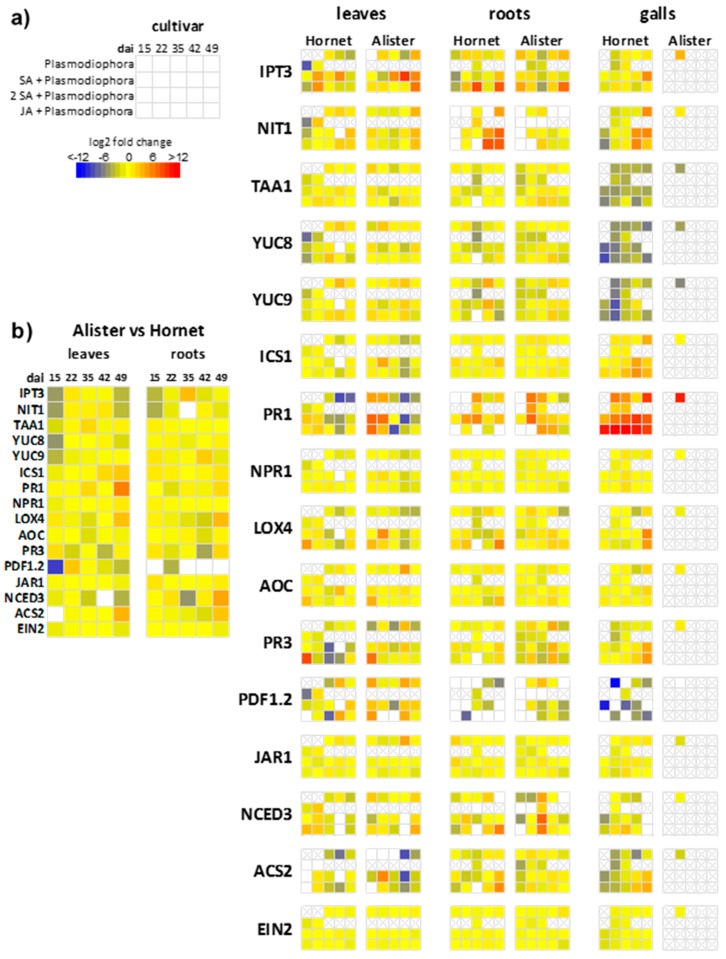
Expression patterns of hormone-related genes. Mean qRT-PCR values of the experimental variants (for description, see Figure 2) are related to (**a**) control at each time point (leaves are related to leaves of the respective cultivar, roots, and galls are related to roots of the respective cultivar). Results are organized as in the top scheme. Non-analyzed variants are crossed. White fields correspond to non-detected expression. (**b**) Control Alister plants (leaves or roots) are compared with control Hornet plants. Mean values ± SD and statistical evaluation are shown in Table S3.

Gene expression was followed in the later stages of infection between 15–49 dai (Figure 7). The expression of the CK biosynthetic gene *isopentenyltransferase IPT3* was diminished in infected leaves in Hornet between 42–49 dai, and in Alister at only 42 dai. Down-regulation of the CK plastid biosynthetic pathway might reflect a promoted senescence of infected leaves in the later stages of infection.

This assumption is in accordance with enhanced *IPT3* expression after double SA application, which promoted stress tolerance. After JA application, *IPT3* expression had generally similar levels as in the *P. brassicae*-infected variant. In roots of both cultivars, *IPT3* expression was generally low, with exception of JA-treated plants (in Hornet, 35 dai and 49 dai, in Alister, 49 dai). The *IPT3* expression was slightly enhanced in galls.

#### 2.5.2. Auxin-Related Genes

The expression of four IAA biosynthetic genes was investigated—*nitrilase 1* (*NIT1*; indole-3-acetonitrile pathway), *tryptophan aminotransferase* (*TAA1;* indole-3-pyruvic pathway) and *indole-3-pyruvate monooxygenase 8* and *9* (*YUC8* and *YUC9* via indole-3-pyruvic acid). *P. brassicae* infection led to their mild transient suppression in Alister leaves, their up-regulation was found in Hornet leaves from 35 dai on.

In Hornet and Alister roots, a contrasting behavior was observed in the expression levels of *NIT1,* the auxin biosynthetic gene that is most commonly involved in the response to infection. In the sensitive cultivar, the gene expression was higher than in the resistant one, further increasing especially after JA application. A similar response was found in Hornet galls.

#### 2.5.3. Salicylic Acid-Related Genes

The expression of the SA biosynthetic gene *isochorismate synthase 1* (*ICS1*) was higher in leaves of Alister than in those of Hornet already at control conditions. This was associated with the elevated expression of the SA-inducible gene *PR1*. During later stages of *P. brassicae* infection, a suppression of *ICS1* expression was detected, especially in Alister. However, the *PR1* expression in Alister was still enhanced within 15 and 22 dai. Double SA application resulted in the stimulation of *PR1* expression in Alister leaves 15 and 22 dai, but only a moderate elevation was observed in Hornet ones. JA application was generally associated with the down-regulation of *ICS1*. The expression of the central component of the SA signaling pathway, *NPR1*, exhibited only minor changes during infection, and after hormone treatment in leaves (with a tendency to decrease in Alister).

Alister exhibited higher *ICS1* expression under control conditions, and also in roots compared to Hornet. *P. brassicae* infection was associated with a slight elevation of *ICS1* expression in Hornet, and somewhat more in Alister. Also *PR1* expression was enhanced in both cultivars (till 35 dai). Double SA treatment resulted in the up-regulation of *PR1* expression in both cultivars. After JA treatment *ICS1* profiles resembled those of infected plants, *PR1* expression was often under the detection limit. In roots, the *NPR1* expression did not show any significant change.

In galls, a mild elevation of *ICS1* expression was detected in Hornet, a moderate increase being found after double SA and JA treatments (due to the lack of galls, the corresponding samples could not be evaluated in Alister). *P. brassicae* infection was associated with a profound stimulation of *PR1* expression over time. Double SA treatment gradually up-regulated *PR1* expression in Hornet. Also JA treatment coincided with high *PR1* expression.

#### 2.5.4. Jasmonic Acid-Related Genes

The expression of the JA biosynthetic gene *lipoxygenase 4* (*LOX4*) decreased upon *P. brassicae* infection in leaves of both cultivars, more in Alister. In contrast, a mild elevation of the other gene encoding a JA biosynthetic enzyme—*allene oxide cyclase* (*AOC*) was observed in the later stages. JA application resulted in the transient up-regulation of both genes in both cultivars 15 dai, possibly via a positive feedback loop. The JA-inducible gene *PR3* was suppressed by *P. brassicae* infection especially in the resistant Alister. Double SA treatment resulted in a substantial decrease of *PR3* expression in Hornet; in Alister, it abolished the effects of infection. JA application led to a strong transient increase of *PR3* expression in both cultivars 15 dai, corresponding well to the transient elevation of JA levels after foliar application. *P. brassicae* infection stimulated *PDF1.2* expression in leaves of both cultivars at the later stages (42–49 dai). The expression of *JA amido synthetase* (*JAR1*), which conjugates JA to isoleucine, was generally not affected by *P. brassicae* infection. JA treatment had in the long-term a negative effect on *JAR1* expression in Alister leaves.

The expression patterns of both *LOX4* and *AOC* followed similar profiles in roots as in leaves after *P. brassicae* infection. Double SA treatment decreased *LOX4* transcript levels in Alister roots 15 dai. JA had generally a positive effect on *LOX4* expression in roots. *PR3* expression showed a slightly decreasing trend in roots after *P. brassicae* infection. Both SA treatments led to the down-regulation of *PR3* in Alister roots over a prolonged period. *P. brassicae* infection was associated with the decrease of *PDF1.2* expression in Hornet roots. The expression of *JAR1* was up-regulated in Hornet and down-regulated in Alister 15 dai after infection in all infected variants except for JA treatment.

*LOX4* and *AOC* expression were up-regulated in galls of Hornet 49 dai, especially after double SA and JA treatments, but the expression of the former gene was reduced in Alister galls. *PR3* and *PDF1.2* expression was low in Hornet galls; however, the expression of *PR3* increased after double SA and JA applications during later stages. *JAR1* expression was lower in Alister than in Hornet after *P. brassicae* infection.

#### 2.5.5. Abscisic Acid-Related Genes

The expression of the gene encoding the rate-limiting step in ABA biosynthesis, *9-cis-epoxycarotenoid dioxygenase* (*NCED3*), showed no distinct trend in the expression under control conditions in leaves of both cultivars. Similarly, no consistent trend was observed after *P. brassicae* infection with or without SA treatment. A mild elevation was found after JA application in leaves 15 dai, which was more profound in Hornet than in Alister. In roots, also a similar variable situation concerning the *NCED3* expression was found. After JA application a small elevation was found between 35–49 dai. In addition, the expression of *NCED3* was elevated in galls of JA-treated plants of the cultivar Hornet.

#### 2.5.6. Ethylene-Related Genes

The expression of the ethylene biosynthetic gene *1-aminocyclopropane-1-carboxylic acid synthetase2* (*ACS2*) was decreased by *P. brassicae* infection in leaves and roots of both cultivars and the effect was partially diminished by both SA and JA treatments. *ACS2* expression was higher in roots of the cultivar Hornet compared to Alister. In addition, *ACS2* expression was up-regulated 42 and 49 dai in Hornet galls treated with JA. A slightly lower effect was found in the case of the double SA application. The expression of a crucial component of ethylene signaling pathway *EIN2* was not significantly affected by any treatment either in leaves or in roots.

## 3. Discussion

### 3.1. The Early Response is Characterized by Changes in Growth Promoting Hormones

**Figure 8:**
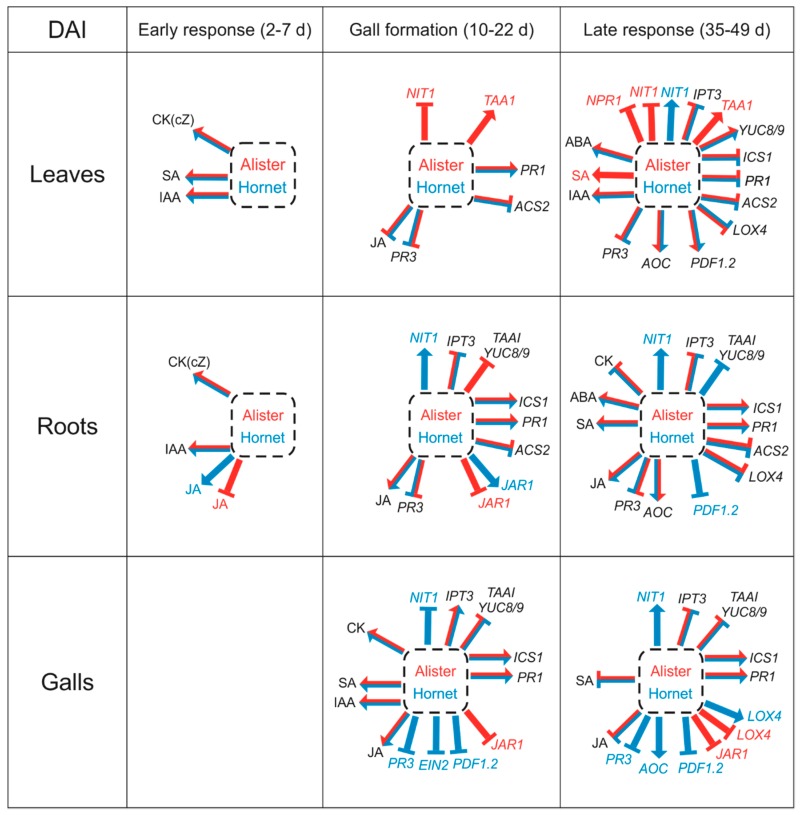
Summary of the *Plasmodiophora brassicae* impact on *Brassica napus* plants during infection progression. Up- or down-regulation of phytohormone contents and gene expression levels in Alister (specific response—red letters and arrows) and Hornet (specific response—blue letters and arrows) leaves, roots and galls detected during early phase (2–7 dai), gall formation (10–22 dai), and late phase (35–49 dai). Response common for both cultivars—black letters.

Colonized cells were demonstrated to exhibit a stimulation of cell division and elongation (e.g., [11]). This is in agreement with the elevation of CKs in roots during the early response to *P. brassicae* infection (2–10 dai; Figure 2, Table S2). The summary of the impact of *P. brassicae* infection on hormone and gene expression data is shown in Figure 8. Active CKs are partially produced by the pathogen, as indicated by the conversion of a precursor to an active CK [9], and by the occurrence of two putative *IPT* genes in the genome [21]. In accordance with previous data on *Brassica rapa* [22] or *Arabidopsis thaliana* [11], we detected the increase of active CKs, predominantly caused by *cis*-zeatin, already 2 dai in roots and leaves of both cultivars (Figure 2). In the disease-sensitive cultivar Hornet, the elevation of the CK content was maintained for 10 days in both organs, while in the resistant cultivar Alister, it dropped to the control level 5 dai, and further decreased below the controls in leaves 7 dai, and in roots 10 dai. Transient CK elevation is in accordance with the up-regulation of several *IPT* genes during the first phase of infection in inoculated roots reported for *Brassica rapa* (Chinese cabbage [23]). The CK down-regulation seems to be part of plant defense, as plants over-expressing CK degrading enzyme cytokinin oxidase/dehydrogenase are more resistant to the clubroot disease [10]. This effect can be interpreted as the reduction of the signals involved in cell division, because the elevated CKs trigger the local re-initiation of cell cycle progression in the root cortex, as indicated by up-regulation of cyclin *CYCB1;1* expression described by Devos et al. [11].

Stimulation of cell division requires also the presence of auxin. The elevated content of IAA was reported in *Brassica campestris* 10 dai [24]. In *Brassica napus,* up-regulation of IAA was detected after 3 dai, to a higher extent in roots [25]. In *Arabidopsis*, an enhanced response of auxin-related genes was observed from 6 dai [11]. Due to the importance of active polar auxin transport [11], it seems that the host auxin needs to be moved to the site of infection. In addition, the signaling pathways responsible for auxin also need to be functioning [26,27]. Our analysis revealed a transient elevation of IAA contents in leaves 2 dai in Alister, and 2–5 dai in Hornet (Figure 3). In roots, IAA content was promoted between 2–7 dai in Alister, and from 2 dai to 15 dai in Hornet. The stronger induction in the susceptible cultivar indicates a prominent role for IAA in disease progression.

Not many changes in defence-related hormones were detected during the early response (Figure 4, Figure 5, Figure 6 and Figure 8). The content of SA in roots was similar as in the controls (Figure 4), while the content of JA was transiently elevated in Hornet roots between 2–5 dai, and in Alister at 7 dai (Figure 5). This is in accordance with the results of Irani et al. [27], who showed that *P. brassicae* stimulated the expression of JA related genes in *A. thaliana*, but not genes related to SA. It seems that the onset of *P. brassicae* infection is associated with colonization of the root tissue, but still without visible symptoms [28].

### 3.2. Differences in the Gall Formation of the Susceptible Cultivar Hornet and Resistant Cultivar Alister

The later phase of *P. brassicae* infection can be characterized by gall formation, which was more profound in the susceptible cultivar Hornet (Table 1). During this stage, the CK content in leaves was higher in Alister (at 15–42 dai) than in Hornet (Figure 2, Table S2) which may reflect the better physiological state of the more tolerant cultivar. CK down-regulation was found in the roots of both cultivars during the later stage. This is in agreement with the results of Malinowski et al. [12], who reported that CKs were diminished in *A. thaliana* after infection (16 dai). In galls of both cultivars, however, high CK levels were detected 22 dai. 

In addition, auxin might also be responsible for enlarged cells during the development of large sporulating plasmodia and resting spores. In roots, the susceptible cultivar Hornet exhibited increased IAA content until 15 dai (Figure 3). In both cultivars, the IAA level was elevated in galls 22 dai (to a higher extent in Hornet), which might be related to cell expansion governed by auxin. In Hornet galls, *NIT1* transcription was up-regulated 35 dai, and especially at 49 dai (Figure 7). Our data are in accordance with several reports that describe the up-regulation of transcription of *nitrilase* isoforms in clubroot tissues compared to controls in different host plants such as *Brassica rapa* (turnip [29], Chinese cabbage [30]), or *Brassica juncea* [31], and specific expression of *nitrilase 1* and *2* genes in developing galls of *Arabidopsis thaliana*, as well as with the up-regulation of myrosinases and glucosinolate metabolism [13,32,33]. The difference between IAA levels and the expression of IAA-biosynthetic genes may be given by the up-regulation of the expression of *GH3* genes (encoding auxin conjugating enzymes), as was shown in the case of *Arabidopsis thaliana* [26]. It may indicate that plant defense includes IAA conjugation, i.e., the inactivation of the excess of IAA involved in gall formation.

SA is the key hormone in plant combat with biotrophic pathogens. In our work, an SA increase in roots was more profound in Alister (35–49 dai) than in Hornet, where it occurred later (42–49 dai; Figure 4). In Alister galls, SA level was enhanced at 22 and 42 dai, while its enhancement was delayed in Hornet (only between 42–49 dai). The expression of SA biosynthetic gene *ICS1* was higher in Alister roots between 35–42 dai, whereas its expression was lower in Hornet root galls (Figure 7). This supports the observation that the induction of defence-related responses in susceptible infected plants is of limited extent and duration [28]. In *A. thaliana*, an up-regulation of the corresponding *ICS1* transcript was reported at 21 but not 28 dai [17]. When the responses to *P. brassicae* infection were followed in the resistant genotype *Brassica macrocarpa* Guss. and in the susceptible genotype *Brassica oleracea* var. *italica* [34], the SA signal transduction was up-regulated mainly in the resistant genotype, in comparison with the susceptible one. Only minor differences were observed in the expression of the central component of the SA signaling pathway *NPR1* (Figure 7). Regulation of its activity seems to be realized predominantly by oligomerization of the resulting protein, and subsequent differential cellular localization. *PR1* expression was up-regulated in Hornet roots 35–49 dai, and in Alister already 22–42 dai (Figure 7). This is in agreement with its role as a marker for SA-dependent defense (e.g., [35]). Constitutive up-regulation of *PR1* expression in the *cpr1* mutant of *A. thaliana* resulted in more clubroot resistant plants [17]. The experiments with SA deficient/insensitive and constitutively active mutants indicate that SA is very important, but not sufficient in defense to *P. brassicae* [17].

During the later stages, the JA content was elevated in the roots of both cultivars (Figure 5), which indicates that JA might not play a role in the defense of the resistant cultivar to the clubroot disease. JA content was especially high during the gall initiation, occurring around 22 dai. As JA is able to induce nitrilase activity, there could be a link between jasmonate and auxin metabolism, and not so much of a signal for plant defense [13,36].

The gene for the rate-limiting enzyme of ethylene biosynthesis, *ACS2,* exhibited lower expression in roots and galls of the susceptible cultivar Hornet compared to the resistant Alister (Figure 7). Knaust and Ludwig-Müller [37] reported for *A. thaliana* that the expression of another gene for the ethylene biosynthetic enzyme *ACO2* was up-regulated throughout the whole experimental period of 10 to 28 dai. However, the expression of a crucial component of the ethylene signaling pathway *EIN2* was not significantly affected in the two *Brassica* cultivars (Figure 7). Ethylene signaling mutants of *A. thaliana* were more susceptible to *P. brassicae* infection, thereby making larger galls at a lower infection pressure [37]. The authors concluded that ethylene might be needed to restrict gall size under normal conditions.

### 3.3. The Impacts of Salicylic Acid and Jasmonic Acid Applications

Taking into account that SA is a crucial hormone in the response to biotrophic pathogens, we tested the effect of exogenous SA applied before infection or twice before infection and 15 dai, when the gall formation starts (Figure 1). The impact of exogenous SA in the fight with the clubroot pathogen is dependent on the time point and method of administration as well as on the plant material. Agarwal et al. [38] reported that a very early incubation with SA significantly reduced the clubroot symptoms of *A. thaliana*, which was confirmed for *Brassica oleracea* (broccoli [39]). However, a later treatment of *A. thaliana* plants with SA during infection had no effect on disease severity [17]. We found that exogenous SA was rather quickly metabolized, as already two days after its application of only a moderate amount of the hormone was left (Figure 4). At the time point of the second foliar spraying 15 dai, a very high peak of SA was detected 2 h after application. SA metabolism may be partly due to the host plant tendency to re-establish hormone homeostasis disturbed by the application of the hormone, or it may be a part of the strategy of the pathogen to down-regulate SA levels, and thus suppress plant defense. Indeed, *P. brassicae* was reported to have high SA-methyltransferase activity, creating an SA transport form, which can be translocated from the galls to the upper plant parts where it is emitted [40]. Consequently, *A. thaliana* plants overexpressing the methyltransferase gene were more susceptible to clubroot [41,42]. A similar mode of action was observed in the case of *Pseudomonas syringae*, which produces coronatine, a compound structurally similar to JA-isoleucine. Coronatine induces the JA signaling pathway, which leads to the suppression of the SA pathway [18]. Also, SA glucosylation might be considered in the SA deactivation [43]. In our experiments, SA application before infection had a negligible effect on gall formation. However, SA application at the onset of gall formation prevented disease development in the more resistant cultivar Alister (Table 1), indicating that in Alister, the defense is already partially working, and therefore, only small amounts of SA increase resistance.

The fact that the JA-metabolic mutant *jar1*, which is unable to produce the active JA metabolite JA-isoleucine, is more sensitive to *P. brassicae* infection, indicates that JA signaling is also involved in the response to *P. brassicae* infection [15]. Nevertheless, exogenous application of JA at 15 dai promoted gall formation in Hornet, but also in the more resistant cultivar Alister (Table 1). Similarly, the partially resistant *Arabidopsis* ecotype Bur was reported to activate after infection of the SA pathway, while the sensitive *Arabidopsis* ecotype Col-0 activated the JA pathway [15]. When more virulent pathotype of *P. brassicae* (isolate e_2_ instead of eH isolate) was used, even Bur did not activate the SA pathway [16]. These data indicate a complex cross-talk among these two pathways which depends on the mutual ratio of their activities.

### 3.4. Organ Specificity—Leaves vs. Roots and Galls

Shoot/root communication is fast and intensive within the plant. This can be demonstrated by changes of leaf hormone levels upon attack by soil pathogens as well as by elevation of SA or JA in roots after their foliar application. SA has been proven to be the key hormone in defense against biotrophs. In some cases, when exogenous SA did not decrease clubroot growth; it diminished at least the stress-induced inhibition of shoot growth [17]. Thus, the (partial) preservation of active CK and auxin content in leaves as shown for both cultivars (Figure 3) might be a measure of the shoot fitness, and not the consequence of the pathogen impact, as is seen in roots. In the latter, the regulation of CK and auxin levels (including the degradation with cytokinin oxidase/dehydrogenase CKX or conjugation with GH3) seems to reflect the combat between the pathogen and plant. Hormone homeostasis in leaves might either be maintained through transport, degradation, or conjugation, or through de novo synthesis. Up-regulation of transcripts for auxin biosynthesis genes in both cultivars, such as *NIT1* and the two *YUC* genes, indicates the stimulation of IAA synthesis (Figure 7).

The modulation of SA and JA content in leaves reflects the intensive activation of the defense, also in organs that are not directly exposed to the pathogen. In our study, the SA level was increased in Alister leaves between 35–49 dai, while in Hornet leaves, the increase occurred earlier, being maintained until the end of the experimental period (between 15–49 dai; Figure 5). In the roots, the SA level increased earlier, and to a higher extent in the resistant than in the susceptible cultivar. Methylation of SA by the protist’s methyltransferase could result in a form that is more readily transported into the leaves, as experiments from *A. thaliana* indicate [40]. The methylester of SA can be converted back to free SA in the leaves using specific esterases. The role of such esterases and methyltransferases for SA metabolism of *B. oleracea* during clubroot infection was demonstrated [44]. Not only in the roots, but also in the leaves, differentially expressed genes were found that could be involved in SA metabolism (Figure 7).

In the case of JA, the situation might be a bit different. While we found that the JA levels were up-regulated in leaves of the cultivar Alister only (15–35 dai), the expression of the JA biosynthetic gene *LOX4* was down-regulated in leaves of both cultivars (Figure 5 and Figure 7).

Finally, the fitness of the plant is also dependent on the water status. In roots, the content of ABA was enhanced in both cultivars during later stages of the infection, a bit higher in the susceptible Hornet than in the resistant Alister cultivar (Figure 6), which confirmed earlier data where ABA was also increased in *Brassica* galls at a later stage of disease development [22]. Ludwig-Müller [28] proposed, based on the up-regulation of many transcripts related to drought stress during later stages of the clubroot disease, a strong influence of ABA. The drought stress could be attributed to the destroyed root tissues that include the vasculature. In our experiments, the content of ABA was enhanced in leaves and roots of both cultivars at 42 and 49 dai (Figure 6). ABA has obviously a dual role in the regulation of stomata aperture and stimulation of the expression of stress-related genes. However, the ABA elevation in leaves seems to be rather a consequence of impaired water transport (leaf wilting) than modulation of the transcriptome.

## 4. Materials and Methods 

### 4.1. Biological Materials and Pathogen Inoculation

The susceptible cultivar of oilseed rape (*Brassica napus* subs. *napus*) Hornet and the clubroot resistant cultivar SY Alister, as well as *Plasmodiophora brassicae* pathotype 6 (according to [45]) isolate were used. Seeds of oilseed rape were planted in pots in sterile potting soil with standard regime (21 °C and 12 h light) and fertilization. Plants were inoculated by *P. brassicae* at the age of 10 days, except control plants. Resting spores were extracted from galled root tissue by the method of Tewari et al. [46] with some modifications, as described by Strelkov et al. [47]. Briefly, 4 g fresh weight (FW) of clubbed roots were ground in 50 mL of distilled water with a blender. The suspension was then filtered through six layers of gauze. The spore suspension was counted in a Bürker chamber according to Neubauer (Blaubrand, Wertheim, Germany), and the resting spore concentration was adjusted to a final concentration of 10^7^ spores/mL. The spore suspension was used for inoculation of the plant material. Seedlings were inoculated by pipetting 4 mL of spore suspension to each plant.

### 4.2. Experimental Design

The experiments were conducted in 2016 and 2017. The experiments included the determination of gall formation, analysis of the levels of phytohormones, and determination of the expression of phytohormone-related genes. The experimental variants included (a) control: susceptible cultivar Hornet and resistant cultivar Alister, not inoculated, (b) plants inoculated by *P. brassicae*, (c) inoculated plants treated once by foliar spray with 1 mM SA (in 0.05% ethanol/water with 0.01% Tween 20) at the day of inoculation, (d) inoculated plants treated by foliar spray with 1 mM SA (in 0.05% ethanol/water with 0.01% Tween 20) at the day of inoculation and 15 dai (chosen because of the beginning of gall formation), (e) inoculated plants treated with 1 mM JA (in water with 0.01% Tween 20) 15 dai. The scheme of the experiment is shown in Figure 1.

The influence of the infection on phytohormone levels was determined in the intervals of 2, 5, 7, 10, 15, 22, 35, 42, and 49 dai; five plants from each variant were taken. The samples for phytohormone and gene expression analyses were collected from roots and leaves, in the case of infected plants also from galls (when formed). At 15 dai, the samples were taken 2 h after exogenous hormone treatment. The samples were immediately frozen in liquid nitrogen and stored at −80 °C. At the same time, the roots were evaluated for gall formation.

### 4.3. Disease Assessment

Disease assessment was performed for each sampling. Plants were removed from the pots and washed with water. The roots were evaluated for clubroot severity on a 0–3 scale [48], where: 0—no galling, 1—a few small galls, 2—moderate galling on the main and lateral roots, and 3—severe galling, the root was totally deformed. The Disease Index (DI) was calculated for each differential host using the formula of Horiuchi and Hori [49] as modified by Strelkov et al. [47]:

(1) $$\mathrm{DI}(\%)=\frac{\sum (n\times 0+n\times 1+n\times 2+n\times 3)}{N\times 3}\times 100$$

where ∑ is the total sum, *n* is the number of plants in the class, *N* is the total number of plants, and 0, 1, 2, 3 are the symptom severity classes.

### 4.4. Phytohormone Analysis

Frozen samples (ca. 50 mg FW) were homogenized and extracted with cold (−20 °C) methanol/water/formic acid (15/4/1, *v*/*v*/*v*) as described previously [50,51]. The following isotope-labelled internal standards (10 pmol/sample) were added: ^13^C_6_-IAA (Cambridge Isotope Laboratories); ^2^H_4_-SA (Sigma-Aldrich); ^2^H_3_-PA, ^2^H_3_-DPA (NRC-PBI); ^2^H_6_-ABA, ^2^H_5_-JA, ^2^H_5_-transZ, ^2^H_5_-transZR, ^2^H_5_-transZ7G, ^2^H_5_-transZ9G, ^2^H_5_-transZOG, ^2^H_5_-transZROG, ^2^H_5_-transZRMP, ^2^H_3_-DZ, ^2^H_3_-DZR, ^2^H_3_-DZ9G, ^2^H_6_-iP, ^2^H_6_-iPR, ^2^H_6_-iP7G, ^2^H_6_-iP9G, ^2^H_6_-iPRMP (Olchemim). Phytohormones were separated with a reverse-phase cation exchange SPE column (Oasis-MCX, Waters) into the acid fraction by elution with methanol (auxins, ABA, SA, JA), and into the basic fraction by elution with 0.35 M NH_4_OH in 60% methanol (CKs). Fractions were analyzed using HPLC (Ultimate 3000, Dionex) coupled to a 3200 Q TRAP hybrid triple quadrupole/linear ion trap mass spectrometer (Applied Biosystems). Hormone quantification was performed by the isotope dilution method with multilevel calibration curves (r^2^ > 0.99). Data processing was performed with the Analyst 1.5 software package (Applied Biosystems).

### 4.5. Quantitative RT-PCR

Samples from leaves and roots were homogenized by cooled ball mill MM301 (Retsch) at 25 Hz, 2.5 min. Gall samples were homogenized with mortar and pestle in liquid nitrogen. RNA was isolated using RNeasy Plant Mini Kit (QIAGEN). Traces of DNA were eliminated using DNase I recombinant (Roche Applied Science). Total RNA was converted to complementary DNA (cDNA) according to the protocol of the M-MLV Reverse Transcriptase (RNase H Minus, Point Mutant, Promega) for the first strand cDNA synthesis. Oligo-dT primers and the Protector RNase Inhibitor (Roche Applied Science) were added to the reaction. cDNA was 20× diluted with RNase-free water. 2.5 μL of each sample was mixed with the LightCycler 480 DNA SYBR Green I Master (Roche Applied Science) and 500 nM of primers to a final volume 10 μL. qPCR was performed by the Light Cycler 480 (Roche Applied Science). Primer annealing temperature was 60 °C (10 s), the elongation (at 72 °C) lasted 10 s. Actin *ACT2* was selected as the reference gene. The relative content of RNA in samples was calculated by the ddCt method [52]. Primers were selected from [53,54,55,56,57,58,59]. Other primers were designed according to the NCBI database [60] using Primer3Plus [61]. The quality of primer pairs was verified by AlleleID (PREMIER Biosoft), and the probability of folding secondary structures was predicted with mfold [62]. The list of primers is presented in Table S1. In the case of the cultivar Alister, gene expression in galls (except 22 dai of *P. brassicae* infection) could not be evaluated, as the amount of the material was limited, and thus, only hormone analysis was done.

### 4.6. Statistic Analysis

Four replicate samples of leaves and roots (*n* = 4) and 2–4 samples of galls (in dependence on gall formation) were analyzed. Results from hormonal and RT-qPCR analyses were evaluated by two-sample Student’s *t*-test with the software PAST 3.01.

## 5. Conclusions

A comparison of hormonal pools under control conditions revealed rather minor differences between Alister and Hornet (except mildly elevated SA content in the resistant cultivar; Figure 8). These differences might reflect a variance among cultivars, thus it seems that it is not possible to distinguish the resistant ones by screening of non-infected plants. *P. brassicae* infection was associated with the early elevation of CKs and auxin in the leaves and roots of both cultivars. In Alister, the CK content in roots was down-regulated quite early. This cultivar exhibited only limited gall formation. Nevertheless, galls of both cultivars had at the beginning of their formation high levels of CKs and IAA (together with SA and JA). SA was increased during later stages of infection, but to a somewhat earlier time point in Alister. The SA marker gene *PR1* exhibited the highest expression around the beginning of gall formation in leaves, roots, and especially in galls. The JA level was higher in Hornet than in Alister during the whole experiment. The difference between the activation of defense hormone pathways in resistant and sensitive cultivars is in accordance with the impact of SA and JA applications—SA further suppressed gall formation in Alister, while JA mildly promoted gall formation in both cultivars. 

## Figures and Tables

**Table 1:** Disease Index (DI; %) for the susceptible oilseed rape cultivar Hornet and the resistant cultivar Alister in the years 2016 and 2017. *Plasmodiophora*–infected plants; SA + *Plasmodiophora*—infected plants treated once with SA; 2 SA + *Plasmodiophora*—infected plants treated twice with SA; JA + *Plasmodiophora*—infected plants treated with JA.

		Disease Index (%)			
dai	Experimental Variant	Hornet		Alister	
		2016	2017	2016	2017
**10**	*Plasmodiophora*	0	0	0	0
	SA + *Plasmodiophora*	0		0	
	2 SA + *Plasmodiophora*	0	0	0	0
	JA + *Plasmodiophora*		0		0
**15**	*Plasmodiophora*	0	0	0	0
	SA + *Plasmodiophora*	0		0	
	2 SA + *Plasmodiophora*	0	19.0	0	0
	JA + *Plasmodiophora*		55.6		0
**22**	*Plasmodiophora*	100.0	40	6.7	6.7
	SA + *Plasmodiophora*	46.7		6.7	
	2 SA + *Plasmodiophora*	40.0	53.3	0	0
	JA + *Plasmodiophora*		60.0		13.3
**35**	*Plasmodiophora*	100.0	33.3	6.7	0
	SA + *Plasmodiophora*	53.3		13.3	
	2 SA + *Plasmodiophora*	46.7	60.0	0	0
	JA + *Plasmodiophora*		83.3		0
**42**	*Plasmodiophora*	100.0	53.3	40.0	0
	SA + *Plasmodiophora*	86.7		6.7	
	2 SA + *Plasmodiophora*	60.0	58.3	0	0
	JA + *Plasmodiophora*		88.9		3.7
**49**	*Plasmodiophora*		100		0
	SA + *Plasmodiophora*				
	2 SA + *Plasmodiophora*		66.7		0
	JA + *Plasmodiophora*		75.0		0

## Supplementary Materials

Supplementary materials can be found at http://www.mdpi.com/1422-0067/19/12/4024/s1.

## Abbreviations

ABA	abscisic acid
*ACS2*	*1-aminocyclopropane-1-carboxylic acid synthetase 2*
*AOC*	*allene oxide cyclase*
CK cZ	cytokinin *cis*-zeatin
dai	days after inoculation
DI	disease Index
*EIN2*	*ethylene insensitive 2*
IAA	indole-3-acetic acid
*ICS1*	*isochorismate synthase 1*
*IPT3*	*isopentenyltransferase 3*
JA	jasmonic acid
*JAR1*	*jasmonate resistant 1*
*LOX4*	*lipoxygenase 4*
*NCED3*	*9-cis-epoxycarotenoid dioxygenase*
*NIT1*	*nitrilase 1*
*NPR1*	*nonexpresser of PR genes*
*PDF1.2*	*plant defensin 1.2*
*PR*	*pathogenesis-related protein*
SA	salicylic acid
*TAA1*	*tryptophan aminotransferase*
*YUC*	*indole-3-pyruvate monooxygenase*

## References

[1] Dixon G.R. (2009). The occurrence and economic impact of *Plasmodiophora brassicae* and clubroot disease. J. Plant Growth Regul..

[2] Ingram D.S., Tommerup I.C. (1972). The life history of *Plasmodiophora brassicae* Woron. Proc. R. Soc. Lond. B.

[3] Wallenhammar A.C. (1996). Prevalence of *Plasmodiophora brassicae* in a spring oilseed rape growing area in central Sweden and factors influencing soil infestation levels. Plant Pathol..

[4] Ludwig-Müller J., Vos C., Kamal K. (2016). Belowground Defence Strategies in Plants. Belowground Defence Strategies in Plants.

[5] Ludwig-Müller J., Prinsen E., Rolfe S.A., Scholes J.D. (2009). Metabolism and plant hormone action during clubroot disease. J. Plant Growth Regul..

[6] Siemens J., González M.C., Wolf S., Hofmann C., Greiner S., Du Y., Rausch T., Roitsch T., Ludwig-Müller J. (2011). Extracellular invertase is involved in the regulation of clubroot disease in *Arabidopsis thaliana*. Mol. Plant Pathol..

[7] Dekhuijzen H.M., Overeem J.C. (1971). The role of cytokinins in clubroot formation. Physiol. Plant Pathol..

[8] Dekhuijzen H.M. (1981). The occurrence of free and bound cytokinins in plasmodia of *Plasmodiophora brassicae* isolated from tissue cultures of clubroots. Plant Cell Rep..

[9] Müller P., Hilgenberg W. (1986). Isomers of zeatin and zeatin riboside in clubroot tissue—Evidence for *trans*-zeatin biosynthesis by *Plasmodiophora brassicae*. Physiol. Plant..

[10] Siemens J., Keller I., Sarx J., Kunz S., Schuller A., Nagel W., Schmülling T., Parniske M., Ludwig-Müller J. (2006). Transcriptome analysis of *Arabidopsis* clubroots indicate a key role for cytokinins in disease development. Mol. Plant Microbe Interact..

[11] Devos S., Laukens K., Deckers P., Van Der Straeten D., Beeckman T., Inzé D., Van Onckelen H., Witters E., Prinsen E. (2006). A hormone and proteome approach to picturing the initial metabolic events during *Plasmodiophora brassicae* infection on *Arabidopsis*. Mol. Plant Microbe Interact..

[12] Malinowski R., Novák O., Borhan M.H., Spíchal L., Strnad M., Rolfe S.A. (2016). The role of cytokinins in clubroot disease. Eur. J. Plant Pathol..

[13] Grsic S., Kirchheim B., Pieper K., Fritsch M., Hilgenberg W., Ludwig-Müller J. (1999). Induction of auxin biosynthetic enzymes by jasmonic acid and in clubroot diseased Chinese cabbage plants. Physiol. Plant..

[14] Schuller A., Kehr J., Ludwig-Müller J. (2014). Laser microdissection coupled to transcriptional profiling of *Arabidopsis* roots inoculated by *Plasmodiophora brassicae* indicates a role for brassinosteroids in clubroot formation. Plant Cell Physiol..

[15] Lemarie S., Robert-Seilaniantz A., Lariagon C., Lemoine J., Marnet N., Jubault M., Manzanares-Dauleux M.J., Gravot A. (2015). Both the jasmonic acid and the salicylic acid pathways contribute to resistance to the biotrophic clubroot agent *Plasmodiophora brassicae* in *Arabidopsis*. Plant Cell Physiol..

[16] Jubault M., Lariagon C., Taconnat L., Renou J.P., Gravot A., Delourme R., Manzanares-Dauleux M.J. (2013). Partial resistance to clubroot in *Arabidopsis* is based on changes in the host primary metabolism and targeted cell division and expansion capacity. Funct. Integr. Genom..

[17] Lovelock D.A., Sola I., Marschollek S., Donald C.E., Rusak G., van Pée K.-H., Ludwig-Müller J., Cahill D.M. (2016). Analysis of salicylic acid-dependent pathways in *Arabidopsis thaliana* following infection with *Plasmodiophora brassicae* and the influence of salicylic acid on disease. Mol. Plant Pathol..

[18] Zheng X.Y., Spivey N.W., Zeng W.Q., Liu P.P., Fu Z.Q., Klessig D.F., He S.Y., Dong X.N. (2012). Coronatine promotes *Pseudomonas syringae* virulence in plants by activating a signaling cascade that inhibits salicylic acid accumulation. Cell Host Microbe.

[19] Kobelt P., Siemens J., Sacristán M.D. (2000). Histological characterisation of the incompatible interaction between *Arabidopsis thaliana* and the obligate biotrophic pathogen *Plasmodiophora brassicae*. Mycol. Res..

[20] Řičařová V., Kazda J., Baranyk P., Ryšánek P. (2017). Greenhouse and field experiments with winter oilseed rape cultivars resistant to *Plasmodiophora brassicae* Wor. Crop Prot..

[21] Schwelm A., Fogelqvist J., Knaust A., Jülke S., Lilja T., Bonilla-Rosso G., Karlsson M., Shevchenko A., Choi S.R., Dhandapani V. (2015). The *Plasmodiophora brassicae* genome reveals insights in its life cycle and ancestry of chitin synthases. Sci. Rep..

[22] Devos S., Vissenberg K., Verbelen J.P., Prinsen E. (2005). Infection of Chinese cabbage by *Plasmodiophora brassicae* leads to a stimulation of plant growth: Impacts on cell wall metabolism and hormone balance. New Phytol..

[23] Ando S., Asano T., Tsushima S., Kamachi S., Hagio T., Tabei Y. (2005). Changes in gene expression of putative isopentenyltransferase during clubroot development in Chinese cabbage (*Brassica rapa* L.). Physiol. Mol. Plant Pathol..

[24] Ludwig-Müller J., Bendel U., Thermann P., Ruppel M., Epstein E., Hilgenberg W. (1993). Concentrations of indole-3-acetic acid in plants of tolerant and susceptible varieties of Chinese cabbage infected with *Plasmodiophora brassicae* Woron. New Phytol..

[25] Xu L., Ren L., Chen K., Liu F., Fang X. (2016). Putative role of IAA during the early response of *Brassica napus* L. to *Plasmodiophora brassicae*. Eur. J. Plant Pathol..

[26] Jahn L., Mucha S., Bergmann S., Horn C., Siemens J., Staswick P., Steffens B., Ludwig-Müller J. (2013). The clubroot pathogen (*Plasmodiophora brassicae*) influences auxin signaling to regulate auxin homeostasis. Plants.

[27] Irani S., Trost B., Waldner M., Nayidu N., Tu J.Y., Kusalik A.J., Todd C.D., Wei Y.D., Bonham-Smith W.C. (2018). Transcriptome analysis of response to *Plasmodiophora brassicae* infection in the *Arabidopsis* shoot and root. BMC Genom..

[28] Ludwig-Müller J. (2009). Plant defence—What can we learn from clubroots?. Austral. Plant Pathol..

[29] Ishikawa T., Okazaki K., Kuroda H., Itoh K., Mitsui T., Hori H. (2007). Molecular cloning of *Brassica rapa* nitrilases and their expression during clubroot development. Mol. Plant Pathol..

[30] Ando S., Tsushima S., Kamachi S., Konagaya K.I., Tabei Y. (2008). Alternative transcription initiation of the nitrilase gene (*BrNIT2*) caused by infection with *Plasmodiophora brassicae* Woron. in Chinese cabbage (*Brassica rapa* L.). Plant Mol. Biol..

[31] Liu Y., Yin Y.-P., Wang Z.-K., Luo Y.-L. (2012). Expression of nitrilases in *Brassica juncea* var. tumida Tsen in root galls caused by *Plasmodiophora brassicae*. J. Integr. Agric..

[32] Grsic-Rausch S., Kobelt P., Siemens J., Bischoff M., Ludwig-Müller J. (2000). Expression and localization of nitrilase during symptom development of the clubroot disease in *Arabidopsis thaliana*. Plant Physiol..

[33] Ludwig-Müller J., Pieper K., Ruppel M., Cohen J.D., Epstein E., Kiddle G., Bennett R. (1999). Indole glucosinolate and auxin biosynthesis in *Arabidopsis thaliana* (L.) Heynh. glucosinolate mutants and the development of the clubroot disease. Planta.

[34] Zhang X., Liu Y., Fang Z., Li Z., Yang L., Zhuang M., Zhang Y., Lv H. (2016). Comparative transcriptome analysis between broccoli (*Brassica oleracea* var. italica) and wild cabbage (*Brassica macrocarpa* Guss.) in response to *Plasmodiophora brassicae* during different infection stages. Front. Plant Sci..

[35] Lebel E., Heifetz P., Thorne L., Uknes S., Ryals J., Ward E. (1998). Functional analysis of regulatory sequences controlling *PR-1* gene expression in *Arabidopsis*. Plant J..

[36] Ludwig-Müller J., Schubert B., Pieper K., Ihmig S., Hilgenberg W. (1997). Glucosinolate content in susceptible and resistant Chinese cabbage varieties during development of clubroot disease. Phytochemistry.

[37] Knaust A., Ludwig-Müller J. (2013). The ethylene signaling pathway is needed to restrict root gall growth in *Arabidopsis* after infection with the obligate biotrophic protist *Plasmodiophora brassicae*. J. Plant Growth Regul..

[38] Agarwal A., Kaul V., Faggian R., Rookes J.E., Ludwig-Müller J., Cahill D.M. (2011). Analysis of global host gene expression during the primary phase of the *Arabidopsis thaliana*-*Plasmodiophora brassicae* interaction. Funct. Plant. Biol..

[39] Lovelock D.A., Donald C.E., Conlan X.A., Cahill D.M. (2013). Salicylic acid suppression of clubroot in broccoli (*Brassicae oleracea* var. italica) caused by the obligate biotroph *Plasmodiophora brassicae*. Austral. Plant Pathol..

[40] Ludwig-Müller J., Jülke S., Geiß K., Richter F., Sola I., Rusak G., Mithöfer A., Keenan S., Bulman S. (2015). A novel methyltransferase from the intracellular pathogen *Plasmodiophora brassicae* methylates salicylic acid. Mol. Plant Pathol..

[41] Bulman S., Richter F., Marschollek S., Benade F., Jülke S., Ludwig-Müller J. (2018). *Arabidopsis thaliana* expressing *PbBSMT*, a gene encoding a SABATH-type methyltransferase from the plant pathogenic protist *Plasmodiophora brassicae*, show leaf chlorosis and altered host susceptibility. Plant Biol. J..

[42] Djavaheri M., Ma L., Klessig D.F., Mithöfer A., Gropp G., Borhan M.H. (2018). Mimicking the host regulation of SA: A virulence strategy by the clubroot pathogen *Plasmodiophora brassicae*. Mol. Plant Microbe Interact..

[43] Vlot A.C., Dempsey D.A., Klessig D.F. (2009). Salicylic acid, a multifaceted hormone to combat disease. Annu. Rev. Phytopathol..

[44] Manoharan R., Shanmugam A., Hwang I., Park J.-I., Nou I.-S. (2016). Expression of salicylic acid-related genes in *Brassica oleracea* var. capitata during *Plasmodiophora brassicae* infection. Genome.

[45] Williams P.H. (1966). A system for the determination of races of *Plasmodiophora brassicae* that infect cabbage and rutabaga. Phytopathology.

[46] Tewari J.P., Strelkov S.E., Orchard D., Hartman M., Lange R.M., Turkington T.K. (2005). Identification of clubroot of crucifers on canola (*Brassica napus*) in Alberta. Can. J. Plant Pathol..

[47] Strelkov S.E., Tewari J.P., Smith-Degenhardt E. (2006). Characterization of *Plasmodiophora brassicae* populations from Alberta, Canada. Can. J. Plant Pathol..

[48] Kuginuki Y., Yoshikawa H., Hirai M. (1999). Variation in virulence of *Plasmodiophora brassicae* in Japan tested with clubroot- resistant cultivars of Chinese cabbage (*Brassica rapa* L. ssp. pekinesis). Eur. J. Plant Pathol..

[49] Horiuchi S., Hori M. (1980). A simple greenhouse technique for obtaining high levels of clubroot incidence. Bull. Chugoku Natl. Agric. Exp. Stn. Ser. E.

[50] Dobrev P.I., Kaminek M. (2002). Fast and efficient separation of cytokinins from auxin and abscisic acid and their purification using mixed-mode solid-phase extraction. J. Chromatogr. A.

[51] Dobrev P.I., Vankova R., Shabala S., Cuin T. (2012). Quantification of abscisic acid, cytokinin, and auxin content in salt-stressed plant tissues. Plant Salt Tolerance. Methods in Molecular Biology (Methods and Protocols).

[52] Livak K.J., Schmittgen T.D. (2001). Analysis of relative gene expression data using real-time quantitative PCR and the 2^−ΔΔCT^ method. Methods.

[53] Joshi R.K., Megha S., Rahman M.H., Basu U., Kav N.N. (2016). A global study of transcriptome dynamics in canola (*Brassica napus* L.) responsive to *Sclerotinia sclerotiorum* infection using RNA-Seq. Gene.

[54] Liang Y., Strelkov S.E., Kav N.N.V. (2009). Oxalic acid-mediated stress responses in *Brassica napus* L.. Proteomics.

[55] Liu F., Li X., Wang M., Wen J., Yi B., Shen J., Ma C., Fu T., Tu J. (2018). Interactions of WRKY 15 and WRKY 33 transcription factors and their roles in the resistance of oilseed rape to *Sclerotinia infection*. Plant Biotechnol. J..

[56] Rodriguez-Sanz H., Solis M.T., Lopez M.F., Gomez-Cadenas A., Risueno M.C., Testillano P.S. (2015). Auxin biosynthesis, accumulation, action and transport are involved in stress-induced microspore embryogenesis initiation and progression in *Brassica napus*. Plant Cell Physiol..

[57] Šasek V., Novakova M., Jindrichova B., Boka K., Valentova O., Burketova L. (2012). Recognition of avirulence gene *AvrLm1* from hemibiotrophic ascomycete *Leptosphaeria maculans* triggers salicylic acid and ethylene signaling in *Brassica napus*. Mol. Plant Microbe Interact..

[58] Song J., Jiang L., Jameson P.E. (2015). Expression patterns of *Brassica napus* genes implicate IPT, CKX, sucrose transporter, cell wall invertase, and amino acid permease gene family members in leaf, flower, silique, and seed development. J. Exp. Bot..

[59] Zhou T., Hua Y., Huang Y., Ding G., Shi L., Xu F. (2016). Physiological and transcriptional analyses reveal differential phytohormone responses to boron deficiency in *Brassica napus* genotypes. Front. Plant Sci..

[60] Pontius J., Wagner L., Schuler G. (2003). The NCBI Handbook.

[61] Untergasser A., Nijveen H., Rao X., Bisseling T., Geurts R., Leunissen J.A. (2007). Primer3Plus, an enhanced web interface to Primer3. Nucleic Acids Res..

[62] Zuker M., Mathews D.H., Turner D.H., Barciszewski J., Clark B.F.C. (1999). Algorithms and thermodynamics for RNA secondary structure prediction: A practical guide. RNA Biochemistry and Biotechnology.

