# Supplementary material for: Hormonal Responses to Plasmodiophora brassicae Infection in Brassica napus Cultivars Differing in Their Pathogen Resistance
Source: Int J Mol Sci. 2018 Dec 13;19(12):4024. doi: 10.3390/ijms19124024 (PMC6321006; doi:10.3390/ijms19124024)
Supplement: Supplementary file 1 [file ijms-19-04024-s001.zip › Table S1-List of primers.docx]

**Table S1:** List of primers for *Brassica napus*. Primers without citation were designed according to the mentioned sequences from NCBI database (Pontius et al., 2003). Primers were designed using Primer3Plus (Untergasser et al., 2007). The quality of primer pairs was verified by AlleleID (PREMIER Biosoft) and the probability of folding secondary structures was predicted with mfold (Zuker et al., 1999).

| **Gene** | **Description** |  | **Primers** | **Source** |
| --- | --- | --- | --- | --- |
| *IPT3* | isopentenyltransferase 3 | F: | gcttccaatcatcgttggaggtt | Song et al. (2015) |
|  |  | R: | tctctagcttcttcgaccattccat |  |
| *ACT2* | actin 2 | F: | ctggaattgctgaccgtatgag | Sasek et al. (2012) |
|  |  | R: | tgttggaaagtgctgaggga |  |
| *LOX4* | lipoxygenase 4 | F: | gttagcgcggatggtgtgat | Joshi et al. (2016) |
|  |  | R: | tgggtcaggaacagccattc |  |
| *EIN2* | ethylene insensitive 2 | F: | ggaaaatgcacaacagcagtca | Liang et al. (2009) |
|  |  | R: | gtccggcctttcctacaagag |  |
| *ACS2* | 1-amino-cyclopropane-1-carboxylate synthase 2 | F: | aggtggtcaaagacttagatag | Sasek et al. (2012) |
|  |  | R: | accgagtcgttgtaagaata |  |
| *PDF1.2* | plant defensin 1.2 | F: | tgccctactttttgctgctc | EE421691 |
|  |  | R: | catgtcgtgctttctcaagg |  |
| *AOC* | allen oxide cyclase | F: | caagcaaaaacccgaggagtt | Liang et al. (2009) |
|  |  | R: | ctggtggcatattgactcgaaa |  |
| *JAR1* | jasmonate resistant 1 | F: | tttgagcaactggtacgg | XM_022718996.1 |
|  |  | R: | cagatcaccagcgtaatgcc |  |
| *PR3* | pathogenesis-related protein 3 | F: | atgcctttatcaatgccgctaa | Liu et al. (2018) |
|  |  | R: | actgtccgtagttgtagttcca |  |
| *ICS1* | isochorismate synthase 1 | F: | caaactcatcatcttccctc | Sasek et al. (2012) |
|  |  | R: | agcgtgacttactaaccag |  |
| *NPR1* | nonexpresser of PR genes 1 | F: | tgctctgttgatcgcgaaac | Liang et al. (2009) |
|  |  | R: | cgcctttggcagctaacttc |  |
| *PR1* | pathogenesis-related protein 1 | F: | catccctcgaaagctcaagac | Sasek et al. (2012) |
|  |  | R: | ccactgcacgggacctac |  |
| *NCED3* | nine-*cis*-epoxycarotenoid dioxygenase 3 | F: | cgatttgccttaccaagtcag | Sasek et al. (2012) |
|  |  | R: | tttatcccttccggtgagaa |  |
| *NIT1* | nitrilase 1 | F: | agataaactggcggaactagc | Zhou et al. (2016) |
|  |  | R: | catgaccttacggtgcttac |  |
| *TAA1* | L-tryptophan-pyruvate aminotransferase 1-like | F: | cgtcacccaataacccagac | Rodriguez-Sanz et al. (2015) |
|  |  | R: | catcttctttgccacctcct |  |
| *YUC8* | yucca 8 | F: | cgagaagtacggtttgaaacgac | XM_013812806.2 |
|  |  | R: | accggcgtcttccctttc |  |
| *YUC9* | yucca 9 | F: | gtcgaaagatcagactgcatagc | XM_013840048.2 |
|  |  | R: | cgatgaactgtcgttttgttggg |  |

**References:**

Joshi, R.K.; Megha, S.; Rahman, M.H.; Basu, U.; Kav, N.N. A global study of transcriptome dynamics in canola (*Brassica napus* L.) responsive to *Sclerotinia sclerotiorum* infection using RNA-Seq. *Gene* **2016**, 590, 57–67, doi: 10.1016/j.gene.2016.06.003.

Liang, Y.; Strelkov, S.E.; Kav, N.N.V. Oxalic acid‐mediated stress responses in *Brassica napus* L. *Proteomics* **2009**, 9, 3156–3173, doi: 10.1002/pmic.200800966.

Liu, F.; Li, X.; Wang, M.; Wen, J.; Yi, B.; Shen, J.; Ma, C.; Fu, T.; Tu, J. Interactions of WRKY 15 and WRKY 33 transcription factors and their roles in the resistance of oilseed rape to *Sclerotinia infection*. *Plant Biotechnol. J.* **2018**, 16, 911–925, doi: 10.1111/pbi.12838.

Pontius, J.; Wagner, L.; Schuler, G. The NCBI Handbook. National Center for Biotechnology Information: Bethesda, USA; 2003.

Rodriguez-Sanz, H.; Solis, M.T.; Lopez, M.F.; Gomez-Cadenas, A.; Risueno, M.C.; Testillano, P.S. Auxin biosynthesis, accumulation, action and transport are involved in stress-induced microspore embryogenesis initiation and progression in *Brassica napus*. *Plant Cell Physiol.* **2015**, 56, 1401–1417, doi: 10.1093/pcp/pcv058.

Šasek, V.; Novakova, M.; Jindrichova, B.; Boka, K.; Valentova, O.; Burketova, L. Recognition of avirulence gene *AvrLm1* from hemibiotrophic ascomycete *Leptosphaeria maculans* triggers salicylic acid and ethylene signaling in *Brassica napus*. *Mol. Plant-Microbe Interact.* **2012**, 25, 1238–1250, doi: 10.1094/MPMI-02-12-0033-R.

Song, J.; Jiang, L.; Jameson, P.E. Expression patterns of *Brassica napus* genes implicate IPT, CKX, sucrose transporter, cell wall invertase, and amino acid permease gene family members in leaf, flower, silique, and seed development. *J. Exp. Bot.* **2015**, 66, 5067–5082, doi: 10.1093/jxb/erv133.

Untergasser, A.; Nijveen, H.; Rao, X.; Bisseling, T.; Geurts, R.; Leunissen, J.A. Primer3Plus, an enhanced web interface to Primer3. *Nucleic Acids Res.* **2007**, 35, W71–W74, doi: 10.1093/nar/gkm306.

Zhou, T.; Hua, Y.; Huang, Y.; Ding, G.; Shi, L.; Xu, F. Physiological and transcriptional analyses reveal differential phytohormone responses to boron deficiency in *Brassica napus* genotypes. *Front. Plant Sci.* **2016**, 7, 221, doi: 10.3389/fpls.2016.00221.

Zuker, M.; Mathews, D.H.; Turner, D.H. Algorithms and thermodynamics for RNA secondary structure prediction: a practical guide. In *RNA Biochemistry and Biotechnology;* Barciszewski J., Clark B.F.C., Eds.; Springer: Dordrecht, Netherlands, 1999; Volume 70, ISBN 978-0-7923-5862-6.
